# The Efficacy and Safety of Elobixibat for the Elderly with Chronic Constipation: A Multicenter Retrospective Cohort Study

**DOI:** 10.1155/2020/9656040

**Published:** 2020-04-28

**Authors:** Akira Tomie, Naohisa Yoshida, Munehiro Kugai, Ryohei Hirose, Osamu Dohi, Ken Inoue, Kotaro Okuda, Takayuki Motoyoshi, Kohei Fukumoto, Yoshikazu Inagaki, Hiroyuki Yoriki, Yutaka Inada, Takashi Okuda, Daisuke Hasegawa, Kiyoshi Ogiso, Takaaki Murakami, Koichi Soga, Rafiz Abdul Rani, Norimasa Yoshida, Yoshito Itoh

**Affiliations:** ^1^Department of Gastroenterology, Saiseikai Kyoto Hospital, Kyoto, Japan; ^2^Department of Molecular Gastroenterology and Hepatology, Kyoto Prefectural University of Medicine, Graduate School of Medical Science, Kyoto, Japan; ^3^Department of Gastroenterology, Akashi City Hospital, Hyogo, Japan; ^4^Department of Gastroenterology, Kyoto Kujo Hospital, Kyoto, Japan; ^5^Department of Gastroenterology, Kyoto City Hospital, Kyoto, Japan; ^6^Department of Gastroenterology, Nara City Hospital, Nara, Japan; ^7^Department of Gastroenterology, Nishijin Hospital, Kyoto, Japan; ^8^Department of Gastroenterology, Otsu City Hospital, Shiga, Japan; ^9^Department of Gastroenterology, Fukuchiyama City Hospital, Kyoto, Japan; ^10^Department of Gastroenterology, Ayabe City Hospital, Kyoto, Japan; ^11^Department of Gastroenterology, Osaka General Hospital of West Japan Railway Company, Osaka, Japan; ^12^Department of Gastroenterology, Japan Community Health Care Organization, Kyoto Kuramaguchi Medical Center, Kyoto, Japan; ^13^Department of Gastroenterology, Omihachiman Community Medical Center, Shiga, Japan; ^14^Gastroenterology Unit, Faculty of Medicine, Universiti Teknologi MARA, Selangor, Malaysia

## Abstract

**Materials and Methods:**

This was a multicenter retrospective cohort study. The subjects were patients aged ≥20 years treated for chronic constipation from May 2018 to November 2019 at 12 related institutions. Patients were divided into ≤74 years and ≥75 years old. Elobixibat at 10 mg/day was prescribed for two weeks. We then analyzed the discontinuation due to ineffectiveness, change of spontaneous bowel movements (SBM), stool consistency, the time until the first SBM, adverse events, and effect-related factors.

**Results:**

There were 140 cases (61 males) evaluated, with an average age of 72.1 ± 13.6 years (≤74 years: 71 cases; ≥75 years: 69 cases). The discontinuation rate was 7.9%. The SBM (times/week) increased from 2.86 to 6.08 (*p* < 0.001). The overall SBM improvement rate was 74.0% (≤74 years: 78.2% vs. ≥75 years: 68.9%, *p* = 0.31; male: 75.0% vs. female: 73.3%, *p* = 0.78). The overall improvement rate of stool consistency was 59.6% (≤74 years: 62.9%, ≥75 years: 56.1%, *p* = 0.42). The time until the first SBM (hours) for those ≤74 years and ≥75 years was 17.2 ± 14.3 and 11.2 ± 8.4 (*p* = 0.04). Adverse event rates for those ≤74 years and ≥75 years were 28.2% and 10.1% (*p* < 0.01). There were no significant effect-related factors for gender, age, and use of laxatives.

**Conclusions:**

Short-period elobixibat is shown to be effective also for the elderly and male.

## 1. Introduction

According to a national survey conducted in 2013, the prevalence of chronic constipation in Japan was 2.6% for male and 4.9% for female. It increases with age for both genders, with the prevalence rate assumed at approximately 10% for those who are ≥75 years old [1]. The prevalence in Western countries has been reported to be approximately 15% throughout the entire age group with the frequency also increasing according to age [2–4]. It has been reported that QOL was lower in patients with chronic constipation compared to healthy individuals, indicating the importance of treatment [5].

In Japan, magnesium oxide and anthraquinolone stimulant laxatives have been widely used for chronic constipation. The guideline for the medical treatment of constipation set by the American College of Gastroenterology proposes lifestyle habit guidance and the administration of osmotic laxatives, similar to the Japanese guidelines [6–8]. In these 10 years, several new drugs for chronic constipation were launched. Lubiprostone, a selective chloride channel activator, and linaclotide, a guanylate cyclase C receptor agonist, were introduced to the market in 2012 and 2017, respectively [9–16]. Elobixibat, a bile acid transporter inhibitor, was launched in 2018 in Japan as a world's first. It increases bile acid influx into the colon by inhibiting bile acid transporters expressed on epithelial cells of the terminal ileum, therefore suppressing bile acid reabsorption, increasing water secretion into the colorectum, and increasing gastrointestinal motility. Some researches had been performed on elobixibat in Sweden and United States around 2011 and followed by several clinical trials in Japan around 2018 [17–23]. However, studies to date have included safety studies with less than 50 cases, and even when more than 100 cases were scrutinized, the average age of subjects was approximately 50 years and 75% or more were female.

In this study, we investigated the efficacy and safety of elobixibat for chronic constipation in ≥100 cases including the elderly and male.

## 2. Materials and Methods

We conducted a multicenter, single-arm, and retrospective cohort study. The subjects were 157 patients with chronic constipation who were prescribed elobixibat, from May 2018 to November 2019, at a total of 12 related institutions (Saiseikai Kyoto Hospital, Kyoto Prefectural University of Medicine, Akashi City Hospital, Kyoto Kujo Hospital, Kyoto City Hospital, Nara City Hospital, Nishijin Hospital, Otsu City Hospital, Fukuchiyama City Hospital, Ayabe City Hospital, Osaka General Hospital of West Japan Railway Company, and JCHO Kyoto Kuramaguchi Medical Center). The eligibility criteria were patients over the age of 20 and suffering from two or more of the following six criteria of chronic constipation under the Rome IV standard: (1) straining, (2) hard stool, (3) residual stool feeling, (4) occlusion feeling, (5) manual bowel movement performed at a frequency of 25% or more of bowel movements, and (6) frequency of bowel movements of <3 times a week [24]. The term “chronic” was defined as “symptoms that have been present for at least 6 months and the criteria for ‘constipation' described above have been met for at least 3 months.” We excluded patients with severe cardiopulmonary, hepatic, or renal disease. We also excluded cases in which patients could not continue taking elobixibat due to factors other than ineffectiveness or side effects. Excluded were also cases at which the data of all three items such as stool frequency, stool consistency, and side effects, were unavailable. Regarding the way of administration of elobixibat, oral administration was initiated at 10 mg once a day before breakfast, but it was possible to reduce the dose to 5 mg once a day before breakfast or increase the dose to 15 mg once a day before breakfast depending on the symptoms. Elobixibat was continued for 2 weeks. This study was conducted based on the World Medical Association Helsinki Declaration and approved by the ethics committee of the representative facility, Saiseikai Kyoto Hospital. This study was retrospective in setting, and an opt-out about the study to the patients was performed in the representative facility.

The evaluation items for this study were the discontinuance rate of elobixibat due to ineffectiveness, adverse events, and the number of spontaneous bowel movements (SBM) one week before and after the administration of elobixibat. We analyzed SBM for the nonelderly aged ≤74 years and elderly patients aged ≥75 years as well as for male and female. Additionally, the improvement rate for SBM was calculated. The number of SBM referred to bowel movements that occurred without a laxative/enema or manual evacuation. The improvement of SBM was defined as the increase of ≥1 bowel movement a week from the baseline and also having ≥3 times bowel movement a week based on a previous report [20]. The changes in stool consistency according to the Bristol stool chart were also analyzed in the nonelderly aged ≤74 years, elderly patients aged ≥75 years, male, and female [25]. The Bristol stool chart is a global standard for evaluating stool shape. The subjects evaluated themselves on a 7-point scale. Types 1 and 2 of this scale are hard stools, whereas types 6 and 7 are loose stools. With respect to the stool consistency, “a change to types 3-5” was considered as having improved. The times until the first SBM after the administration of elobixibat and the presence of SBM within 24 hours were analyzed. Adverse events were also examined. Regarding the analysis of effect-related factors, subjects were divided into two groups, with or without improvement in SBM, in order to conduct a comparative study on gender, age (≤74 years vs. ≥75 years), concomitant use of laxatives, concomitant use of stimulant laxatives, elobixibat dose (5 mg/day vs. 10 mg/day vs. 15 mg/day), the presence of underlying diseases (dyslipidemia, diabetes, hepatic disorder, hypothyroidism, and Parkinson's disease), and other concomitant drugs (antacids, antidepressants, calcium antagonists, Parkinson's disease drugs, narcotics for cancer pain, and ursodeoxycholic acid). We obtained all of patients' information including the evaluation items about elobixibat from the medical records in each hospital. Some facilities utilized a questionnaire after the administration of elobixibat while others did not. It was at the discretion of each facility and doctor.

## 3. Statistical Analysis

The *U* test was used to compare continuous variables for changes in the number of SBM before and after the administration of elobixibat. The improvement rate of SBM, improvement rate of stool consistency, age comparison of the SBM rate within 24 hours, and comparison of background factors depending on whether or not the number of SBM had improved were determined by the chi-squared test and Fisher's exact test (SPSS version 22.0 for Windows; IBM Japan, Ltd., Tokyo, Japan). *p* < 0.05 was considered significant for all statistical analyses.

## 4. Results

We analyzed 140 cases out of the 157 patients. SBM frequency, stool consistency, and side effects were evaluated (Table 1). The gender was 61 males (43.6%) and 79 females (56.4%) with the average age of 72.1 (±13.6) years. In regard to age, 71 cases (50.7%) were ≤74 years old and 69 cases (49.3%) were ≥75 years old. Seventy-six patients (54.3%) used other laxatives in combination with elobixibat. Ninety-two patients (65.7%) continued taking 10 mg elobixibat per day until the second week of administration with 27 patients (19.3%) decreasing the dose to 5 mg, 10 patients (7.1%) increasing the dose to 15 mg, and 11 patients (7.9%) discontinuing. In these 11 cases, 6 were due to adverse events while 5 were due to ineffectiveness.

**Figure 1:**
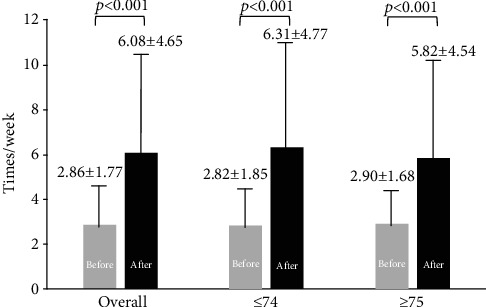
Changes of weekly spontaneous bowel movements after elobixibat with regard to age.

For the 104 evaluated cases of SBM, the frequency of SBM (average ± SD) in the week prior to elobixibat was 2.86 ± 1.77 times/week and the frequency increased to 6.08 ± 4.65 times/week after it (*p* < 0.001) (Figure 1). The frequency (times/week) increased from 2.82 ± 1.85 to 6.31 ± 4.77 in the group of ≤74 years old (*p* < 0.001), and it also increased from 2.90 ± 1.68 to 5.82 ± 4.54 times/week in the group of ≥75 years old (*p* < 0.001), indicating improvements regardless of age.

**Figure 2:**
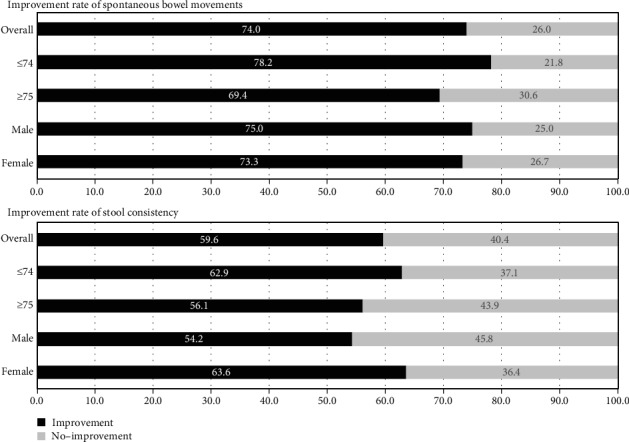
Improvement rate of spontaneous bowel movements and stool consistency after prescription of elobixibat with regard to age and sex.

With respect to the improvement rate in the frequency of SBM, 77 cases were found effective (74.0%) among all 104 evaluated cases (Figure 2). The improvement rate was 78.2% for ≤74 years and 69.4% for ≥75 years with no difference in terms of age (*p* = 0.31). With respect to the effect of gender, the improvement rate was 75.0% for male and 73.3% for female (*p* = 0.78).

Regarding the stool consistency improvement rate, 81 (59.6%) of the 136 cases that could be evaluated showed improvements of stool consistency (Figure 2). It was 62.9% for those ≤74 years and 56.1% for those ≥75 years, indicating no difference by age (*p* = 0.42). With respect to the effect on male and female, it was 54.2% for male and 63.6% for female (*p* = 0.18).

The mean time to first SBM (hours) after taking elobixibat was 14.8 ± 12.6 (Table 2). Regarding age, the time to first SBM was significantly shorter for those ≥75 years compared to those ≤74 years (11.2 ± 8.4 vs. 17.2 ± 14.3, *p* = 0.04). The SBM rate within 24 hours after taking elobixibat was 85 (78.7%) out of the 108 evaluable patients (Table 2). It was 78.3% for ≤74 years and 79.3% for ≥75 years, indicating no difference in terms of age (*p* = 0.92).

Adverse events were observed in 27 (19.3%) of all 140 cases, described as follows: 16 cases (11.4%) of diarrhea, 12 cases (8.6%) of abdominal pain, 4 cases (2.9%) of abdominal distension, and 1 case (0.7%) of nausea. Some cases had more than 2 complaints. The rates for ≥75 years and ≤74 years were 10.1% and 28.2% (*p* < 0.01), respectively. The frequency of adverse events by gender was 16.4% for male and 21.5% for female (*p* = 0.36).

Regarding the analysis of effect-related factors, a comparison was made of background factors, depending on whether SBM had improved or not (Table 3). There was no significant difference with respect to gender, age (≤74 years vs. ≥75 years), concomitant use of laxatives, concomitant use of stimulant laxatives, elobixibat dose, presence of underlying diseases (diabetes, hypothyroidism, Parkinson's disease, dyslipidemia, and hepatic disorder), and the use of concomitant drugs (antacids, Parkinson's disease drugs, antidepressants, calcium antagonists, narcotics for cancer pain, and ursodeoxycholic acid).

## 5. Discussion

The efficacy and safety of elobixibat in ≥100 cases including the elderly and male were verified in this study. The average age of patients was 72.1 ± 13.6 years, and it is the oldest among all elobixibat studies to date. Additionally, regarding gender, our study included 43.6% male subjects (61 cases), being the highest number to date. Regarding the improvement rate of SBM with elobixibat, the rate was 78.2% and there was no significant difference between those ≤74 years old and those ≥75 years old. The rates of adverse events for ≤74 years old and ≥75 years old were 28.2% and 10.1% (*p* < 0.01), respectively. Additionally, there were no significant differences on the improvement rates of SBM, stool consistency, and adverse events between male and female.

In regard to case characteristics, in two studies to verify safety of elobixibat in Sweden and the United States, the number of cases was only 30 and 36, respectively [17, 18]. The only report involving over 100 cases in the United States included 190 subjects; however, the subjects were mainly nonelderly (mean age: 48.1 years) and 90% were female [19]. One report in Japan verified the safety of elobixibat with multiple doses in 59 cases (mean age: 35.4 ± 10.8 years) [21]. Two reports in Japan indicated short-term results from two-week administration: one included 163 patients with an average age of 43.4-46.1 years, among which 88% were female; and the other included 132 patients with an average age of 43.0 years, among which 83% were female [20, 22]. Furthermore, in a Japanese study looking at the long-term results of 52-week treatment, the subjects were 340 patients averaging 43.9 years, among which 83% were female (283 patients), which was biased [20]. Our case's number in thsi study is 140 and included 56.4% females and 69 patients ≥ 75 years old. This is the largest study, which was not biased to either nonelderly or female.

With respect to the efficacy of elobixibat, the improvement rate of SBM was 94.0% at a 10 mg oral dose in a previous Japanese study, which was higher than 74.0% noted in our study which includes the elderly (median age: 72.1 ± 13.6 years, female rate: 56.4%) [20]. We showed that the improvement rates of SBM with regard to age were 78.2% for patients ≤ 74 years old and 69.4% for ≥75 years old. Additionally, we showed that the rates with regard to gender were 75.0% for male and 73.3% for female. The number of SBM after elobixibat administration was 6.40 times/week in the Nakajima et al. study, which was also slightly higher than 6.08 times/week in our study [20]. There was no difference between patients ≤ 74 years old and ≥75 years old. Thus, this is the first report, which showed the efficacy of elobixibat related to age and gender. Our study could indicate that elobixibat is effective for the elderly and male similar to the nonelderly and female. However, in our study, the cases' number was still inadequate, and moreover, it was in a retrospective setting. A large-scale prospective study that is not biased to either age or gender should be performed in the future.

Regarding the first SBM, there was a unique result in our study. Although it was reported that the time (median) to the first SBM was as short as 5.2 hours, the mean time was as long as 14.8 hours in our study [20]. This is probably due to the difference of the characteristics of the patients. The patients in our study were older, and 54.3% of the patients received other laxatives. Thus, patients with a slightly severe constipation compared with those of previous studies were enrolled. Our study also showed that the time in patients aged ≥75 years old was significantly shorter than those aged ≤74 years old, although the SBM/week after elobixibat was slightly smaller in patients aged ≥75 (5.82 ± 4.54) than patients aged ≤74 (6.31 ± 4.77). We suspected that the possible reason for this was that the first SBM was different from the SBM/week affected by many factors including age, sex, laxative, and severity of baseline constipation. Another plausible reason is that the result was due to the retrospective setting of this study. The time was analyzed based on patients' memory, and some might reply with uncertainty. These data should be analyzed prospectively in the future.

With respect to the side effects, two previous studies in Japan reported that the rates of abdominal pain were 26% and 18.8% while those of diarrhea were 5% and 13.0% when 10 mg of elobixibat was administered, whereas the frequency in our study involving the elderly indicated 8.6% for abdominal pain and 11.4% for diarrhea, showing no significant difference, and as such, it should be considered highly safe among both the elderly and the nonelderly [20, 22].

We also examined effect-related factors that were not verified in previous reports. There was no difference to the treatment effect that can be attributed to combination with other laxatives including irritant laxatives, the underlying disease, or the use of concomitant medications. This indicates that the effect was obtained regardless of background factors.

There were several limitations associated with the present study. This was a single-arm retrospective study. There is a possibility that the diagnosis of chronic constipation and the indication of this drug prescription may vary among prescribing physicians.

## 6. Conclusion

In more than 100 clinical cases including the elderly and male, elobixibat for chronic constipation was confirmed to be useful and safe over a short period of time, regardless of age, gender, underlying disease, or concomitant medication.

## Figures and Tables

**Table 1:** Patient characteristics.

Number of cases	140
Gender, *n* (%), male : female	61 : 79 (43.6 : 56.4)
Age, average ± SD	72.1 ± 13.6
Age distribution, *n* (%), ≤74 : ≥75	71 : 69 (50.7 : 49.3)
Laxative combination, *n* (%)	76 (54.3)
Irritant laxative combination, *n* (%)	34 (24.3)
Elobixibat dose (mg), *n* (%), 5 : 10 : 15 : discontinuation	27 : 92 : 10 : 11 (19.3 : 65.7 : 7.1 : 7.9)
Underlying disease, *n* (%)	
Dyslipidemia	42 (30.0)
Diabetes	25 (17.9)
Hepatic disorder	10 (7.1)
Hypothyroidism	6 (4.3)
Parkinson's disease	5 (3.6)
Concomitant medication, *n* (%)	
Antacids	55 (39.3)
Antidepressants	14 (10.0)
Calcium antagonists	11 (7.9)
Parkinson's disease drugs	5 (3.6)
Opioids	4 (2.9)
Ursodeoxycholic acid	3 (2.1)

SD: standard deviation.

**Table 2:** Time to first spontaneous bowel movements and rate of spontaneous bowel movement within 24 hours after prescription of elobixibat.

	Time to first spontaneous bowel movements (hr) (mean ± SD)
Overall (*N* = 95)	14.8 ± 12.6
≤74 (*N* = 56)	17.2 ± 14.3^∗^
≥75 (*N* = 39)	11.2±8.4^∗∗^
	Rate of spontaneous bowel movement within 24 hours
Overall (*N* = 95)	78.7%
≤74 (*N* = 56)	78.3%^∗∗∗^
≥75 (*N* = 39)	79.3%^∗∗∗∗^

SD: standard deviation. ^∗^ vs. ^∗∗^*p* = 0.04, ^∗∗∗^ vs. ^∗∗∗∗^*p* = 0.92.

**Table 3:** The comparison between cases with improvement of spontaneous bowel movements and cases without it.

	With improved bowel movements (*n* = 77)	Without improved bowel movement (*n* = 27)	*p* value
Gender, *n* (%)			
Male	33 (42.9)	11 (40.7)	0.85
Female	44 (57.1)	16 (59.3)	
Age, *n* (%)			
≤74	43 (55.8)	12 (44.4)	0.31
≥75	34 (48.1)	15 (55.6)	
Laxative combination, *n* (%)	43 (44.8)	17 (63.0)	0.52
Irritant laxative combination, *n* (%)	21 (27.3)	6 (22.2)	0.61
Elobixibat dose, *n* (%)			
5 mg	18 (23.4)	4 (14.8)	0.53
10 mg	51 (66.2)	21 (77.8)	
15 mg	8 (10.4)	2 (7.4)	
Underlying disease, *n* (%)			
Dyslipidemia	26 (33.8)	9 (33.3)	0.97
Diabetes	16 (20.8)	5 (18.5)	0.80
Hepatic disorder	3 (3.9)	2 (7.4)	0.46
Hypothyroidism	4 (5.2)	0 (0)	0.23
Parkinson's disease	3 (3.9)	0 (0)	0.30
Concomitant medication, *n* (%)			
Antacids	31 (40.3)	11 (40.7)	0.97
Antidepressants	7 (9.1)	4 (14.8)	0.41
Calcium antagonists	8 (10.4)	2 (7.4)	0.65
Parkinson's disease drugs	3 (3.9)	0 (0)	0.30
Ursodeoxycholic acid	2 (2.6)	0 (0)	0.40

## Data Availability

The patient data used to support the findings of this study are available from the corresponding author upon request. However, some data may be restricted by the institutional review board of Saiseikai Kyoto Prefectural Hospital.
